# Sortilin Is Upregulated in Osteoarthritis-Dependent Cartilage Calcification and Associated with Cellular Senescence

**DOI:** 10.3390/ijms241512343

**Published:** 2023-08-02

**Authors:** Elisabeth Richter, Christoph H. Lohmann, Francesco Dell’Accio, Claudia Goettsch, Jessica Bertrand

**Affiliations:** 1 Department of Orthopaedic Surgery, Otto von Guericke University Magdeburg, 39120 Magdeburg, Germany; elisabeth.richter@st.ovgu.de (E.R.); christoph.lohmann@med.ovgu.de (C.H.L.); 2 William Harvey Research Institute, Queen Mary University London, London EC1M 6BQ, UK; fdellaccio@gmail.com; 3 Department of Internal Medicine I-Cardiology, RWTH Aachen University, 52062 Aachen, Germany

**Keywords:** sortilin, senescence, osteoarthritis, calcification, cartilage, chondrocytes, osteoarthritis

## Abstract

Osteoarthritis (OA) is a chronic joint disease characterized by articular cartilage calcification, loss of articular cartilage, bone changes, pain, and disability. Cartilage calcification is one hallmark of OA and is predominantly caused by basic calcium crystals formed due to an imbalance of the pyrophosphate pathway. Sortilin is a transmembrane protein that contributes to vascular calcification in atherosclerosis by externalizing alkaline phosphatase (ALP)-containing vesicles. Calcification in atherosclerosis and osteoarthritis has been associated with cellular senescence. The aim of this study was to investigate the potential role of sortilin and senescence in osteoarthritis-dependent cartilage calcification. Osteoarthritic cartilage from human knee joints was collected after joint replacement, and samples were analyzed by immunohistochemistry and quantitative RT-PCR analysis. Human chondrocytes were treated with osteogenic medium for up to 21 days to induce calcification. Western blots for sortilin and ALP, as well as an ALP activity assay, were performed. Human chondrocytes were treated with mitomycin C to induce senescence, and sortilin expression was quantified at the protein and gene levels. Sections of knee joints from a murine model of osteoarthritis were stained for sortilin and p16 and analyzed by immunohistochemistry. Treatment of wild-type chondrocytes using an osteogenic medium similar to human chondrocytes was performed. Osteoarthritic cartilage from mouse and human knee joints showed an increased number of sortilin and p16-positive chondrocytes compared to healthy cartilage. This observation was corroborated by increased gene expression of sortilin and p16 in mild and moderate osteoarthritic cartilage samples. To investigate the mechanism of sortilin regulation, human chondrocytes were treated with osteogenic medium to induce calcification. Sortilin protein levels and expression were increased after 7 days of stimulation, whereas ALP levels and activity were upregulated after 21 days of stimulation. Similar observations were made in a murine osteoarthritis model. Mechanistically, senescent chondrocytes induced by mitomycin C showed an upregulation of sortilin and ALP gene expression compared to non-senescent chondrocytes. Our data indicate that sortilin and ALP are upregulated during cartilage calcification, which is associated with chondrocyte senescence and thus might contribute to the pathogenesis of osteoarthritis. Cellular senescence seems to induce sortilin expression.

## 1. Introduction

Osteoarthritis (OA) is the most common chronic disease of the musculoskeletal system. It causes pain and loss of function in the affected joint, predominantly affecting the knee and hip joints [1,2,3]. This leads to impairments in the patients’ quality of life [4]. Age, genetic, metabolic, mechanical, and traumatic factors play a crucial etiological role in regulating the onset of OA [5].

Degenerative and inflammatory processes in the cartilage lead to extracellular matrix (ECM) degradation and remodeling of articular cartilage, including bone layers close to the joint [6]. Activated matrix metalloproteinases (MMPs), particularly MMP-13, and aggrecanases, especially a disintegrin and metalloproteinase with a thrombospondin type 1 motif 4 and 5 (ADAMTS4 and 5), degrade the extracellular cartilage matrix [2,6,7], while chondrocytes undergo hypertrophic differentiation. Hypertrophic chondrocytes secrete increased amounts of collagen 10 instead of collagen 2, depositing calcium crystals in the ECM [6]. This leads to increased calcification of the osteoarthritic cartilage. Cartilage calcification is an obligatory process in OA. As the severity of OA increases, joint calcification also increases proportionally [8]. The calcium crystals in turn stimulate further matrix-degenerating enzymes and aggravate the loss of cartilage elasticity [9].

The deposition of basic calcium crystals (BCP) within cartilage is subject to strict regulation [8]. Excessive crystal formation is physiologically prevented by an extracellular balance between phosphate (Pi) and pyrophosphate (PPi). Shifting the balance in favor of Pi stimulates the formation of BCP crystals [8,10,11]. Alkaline phosphatase (ALP) cleaves PPi to Pi. Therefore, increased enzyme activity increases Pi and associated BCP crystal deposition in OA [10].

Sortilin is a transmembrane protein that acts as a sorting receptor on the cell surface and on the endoplasmic reticulum-Golgi apparatus. It is involved in the transport of various intracellular proteins between the trans-Golgi network, endosome, lysosome, and plasma membrane, as well as the externalization of extracellular vesicles [12,13]. Sortilin is ubiquitously expressed [14]. Sortilin dysregulation is part of the pathogenesis of diseases such as Alzheimer’s disease, type II diabetes mellitus, and atherosclerosis [12,14,15]. Atherosclerosis is characterized by progressive vessel wall calcification [16]. Sortilin promotes atherosclerotic calcifications through intracellular transport and increased excretion of ALP-containing extracellular vesicles [17]. Those extracellular vesicles strongly contribute to the microcalcifications of atherosclerotic plaques [17,18].

Cellular senescence is a physiological process in tissues that protects against malignancy. It is characterized by cell cycle arrest and resistance to apoptosis [19]. The cyclin-dependent kinase inhibitors p16 and p21, among others, contribute to cell cycle arrest [20,21]. Senescent cells secrete proinflammatory cytokines, growth factors, and proteases via exosomes, which is called the senescence-associated secretory phenotype. This phenotype induces senescence in neighboring healthy cells [20,22,23,24]. In aged tissues, senescent, non-functional cells accumulate and promote the development of age-associated diseases. Thus, the contribution of senescence to OA development has been demonstrated previously in several studies [19,21,24,25,26,27].

OA and atherosclerosis share similar mechanisms with respect to tissue calcification. Atherosclerosis, like OA, is a disease of aging and is strongly associated with cellular senescence [28,29,30]. Senescent vascular smooth muscle cells contribute to vascular calcification in atherosclerosis [31]. As in atherosclerosis, BCP deposition in OA is regulated by ALP, and crystal-producing exsomes have also been found in articular cartilage [8,10,32,33,34,35]. Therefore, this work analyzes sortilin expression regulation in the context of calcification in osteoarthritic cartilage.

## 2. Results

### 2.1. Sortilin and Senescence Are Increased in OA Mouse Knees

**Figure 1:**
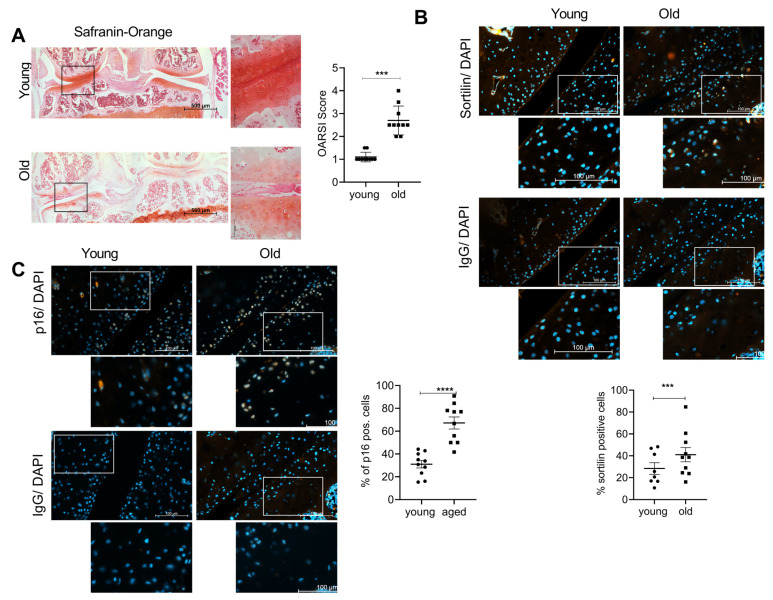
Sortilin and senescence are increased in OA mouse knees. (**A**) Safranin Orange staining of young and old mice (left 100× magnification (scale bar 500 µm), right 400× magnification (scale bar 50 µm)). The OARSI Score of young (8-week-old) and old (45-week-old) mice (young: 1.10 ± 0.211; old: 2.70 ± 0.632; *p* < 0.0001; *N* = 10). (**B**) Sortilin-IF staining of young and old mouse knees (400× magnification (scale bar 100 µm).The white square indicates the amplified area in the picture below. Quantification of sortilin-positive chondrocytes (young: 31.02 ± 10.28; old: 67.17 ± 16.81; *N* = 10). (**C**) p16-IF staining of young and old mouse knees (400× magnification (scale bar 100 µm). The white square indicates the amplified area in the picture below. Quantification of p16-positive chondrocytes (young: 28.42 ± 14.88, old: 41.04 ± 20.46, *p* = 0.0006, *N* < 8). IgG staining was used as a negative control for the specific antibody staining. The statistical evaluation was performed using an unpaired *t*-test. *** *p* < 0.001,**** *p* < 0.0001.

We investigated the expression of sortilin and cellular senescence in natural OA model-aged mice. Representative sections of safranin Orange staining (Figure 1A) and immunofluorescence (IF) staining of sortilin (Figure 1B) and senescence marker p16 (Figure 1C) are shown. The safranin Orange staining clearly shows the hallmarks of OA in the cartilage of old mice. A severe loss of proteoglycans as well as an irregular cartilage surface is present in the old mice (45 weeks), which is absent in the young mice (8 weeks). Old mice significantly showed more abundant signs of OA compared to young mice (*p* < 0.0001). Sortilin-positive cells were significantly more abundant in old mice compared to young mice (*p* = 0.019). At the same time, the number of p16-positive cells was significantly higher in the old mice compared to the young mice (*p* < 0.0001).

### 2.2. Sortilin and Senescence Are Increased in Human OA Cartilage

**Figure 2:**
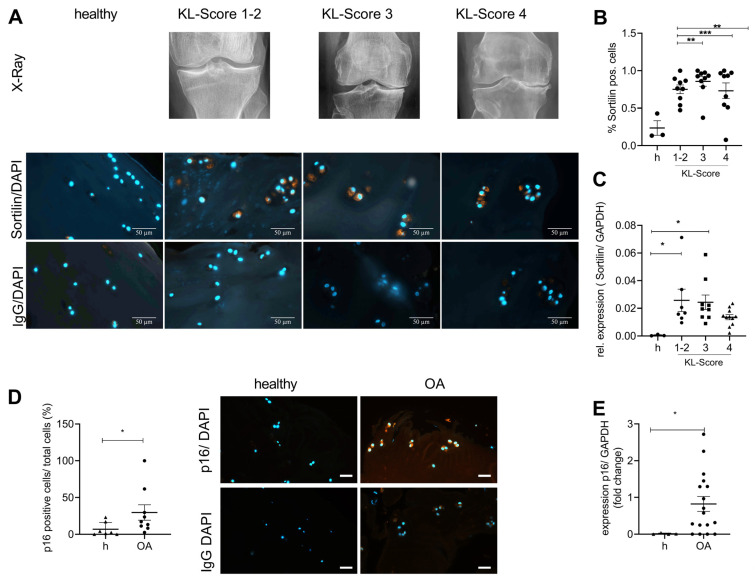
Sortilin and senescence are increased in human OA cartilage. Human cartilage with increasing severity of OA, assessed radiologically by the Kellgren-Lawrence score, was analyzed for sortilin immunostaining and gene expression levels. (**A**) Representative images of sortilin-IF staining of human cartilage are shown (630× magnification, scale bar 50 µm)). (**B**) The quantification of sortilin-positive cells shows a significantly increased number in OA cartilage compared with healthy (h) control (one-way ANOVA: F (3, 26) = 5.568; *p* = 0.004). (**C**) The analyses of sortilin gene expression using qRT-PCR corroborated this result (one-way ANOVA F (3, 26) = 3.236; *p* = 0.038). (**D**) Representative images of p16-IF staining of human cartilage are shown (400× magnification, scale bar, 50 µm). The quantification of p16-positive cells shows a significantly increased number in OA cartilage compared with healthy control (Mann-Whitney test: median: healthy: 2.564; OA: 17.17; *p* = 0.4). (**E**) The analyses of p16 gene expression using qRT-PCR corroborated this result (Mann-Whitney test: Median Healthy: 0.004; OA: 0.57; *p* = 0.04). All pictures were taken at 400× magnification (scale bar 50 µm). IgG staining was used as a negative control for the specific antibody staining. For statistical evaluation, either a one-way ANOVA in the case of comparing more than two groups with Dunett’s post-hoc test or a Mann-Whitney test was performed. The data were analyzed for normal distribution using a Shapiro-Wilk normality test. * *p* < 0.05, ** *p* < 0.01, *** *p* < 0.001.

27 patients were included in this study. The patients were ranked in four groups according to the radiological severity of OA as assessed by the Kellgren-Lawrence Score (KL-score). The patients were divided into mild (KL-score 1–2; *N* = 9); moderate (KL-score 3; *N* = 9); and severe (KL-score 4; *N* = 9) OA. Knee cartilage from three healthy donors who had died without a history of OA was used as a control. Representative X-ray images, sortilin immunostaining, and the respective isotype control are shown in Figure 2A. Sortilin-positive cells are about two-fold increased at all stages of OA severity (Figure 2B). In line with this observation, sortilin gene expression was also significantly increased in mild and moderate OA compared with controls (Figure 2C). In severe OA, sortilin was less upregulated, which was also reflected in fewer sortilin-positive chondrocytes in the immunostaining. Age did not correlate with the number of sortilin-positive chondrocytes in the present cohort (Supplementary Figure S1B: Pearson correlation: r = 0.301; *p* = 0.08). Additionally, no correlation of the patient’s gender with sortilin-positive cells in knee joint cartilage was observed (Supplementary Figure S1B: unpaired t-test, *p* = 0.26). Therefore, age and sex do not seem to correlate with sortilin expression, but the main effect can be attributed to OA changes in cartilage. However, to test whether senescence is increased in OA cartilage, human OA cartilage samples (KL-4) were stained for p16, and the number of positive chondrocytes was counted. The number of p16-positive chondrocytes was significantly increased in OA cartilage (Figure 2D), as was the p16 expression compared to healthy cartilage (Figure 2E).

### 2.3. Sortilin and ALP Are Associated with Increasing Calcification of Human Chondrocytes

**Figure 3:**
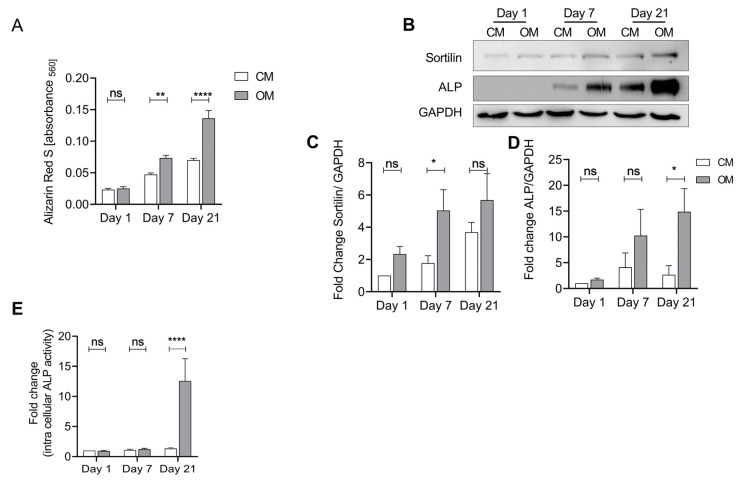
Sortilin and ALP are upregulated in osteogenically differentiated human chondrocytes. Isolated human chondrocytes were treated with osteogenic medium (OM) for up to 21 days to induce calcification. Chondrocyte medium (CM) was used as a control. (**A**) Quantification of eluted Alizarin Red S bound to chondrocytes after treatment with CM and OM to measure calcification. Treatment with OM leads to significantly increased calcification at days 7 and 21, compared with control (CM) (two-way ANOVA F (1, 20) = 29.25; *p* < 0.0001). Furthermore, there is a significant increase in calcification over time (two-way ANOVA F (2, 40) = 22.78; *p* < 0.0001). (**B**) Western blot of sortilin and ALP after osteogenic differentiation. (**C**) Sortilin is significantly increased at day 7 (OM) compared to control (CM). Over time, there continues to be a significant increase in sortilin (two-way ANOVA: F (2, 32) = 13.50; *p* ≤ 0.0001). (**D**) ALP shows a significant increase in protein levels at day 21 in the OM group compared to the control. (two-way ANOVA F (2, 28) = 4.206; *p* = 0.0253). (**E**) ALP activity assay of CM and OM chondrocytes. At day 21, the ALP activity of OM chondrocytes is significantly increased compared to CM chondrocytes (two-way ANOVA: F (2, 38) = 10.97; *p* = 0.0002). For statistical evaluation, a two-way ANOVA with Sidak’s post-hoc test was performed. ns: not significant; * *p* < 0.05, ** *p* < 0.01, **** *p* < 0.0001.

As sortilin function has been associated with tissue calcification and ALP activity, we investigated its role in chondrocytes under calcifying conditions. Therefore, we stimulated isolated human chondrocytes with osteogenic medium to induce calcification. Alizarin Red S was used to quantify the calcification (Figure 3A). We observed a significant increase in Alizarin Red staining upon treatment with osteogenic medium (OM) at days 7 and 21 (*p* < 0.0001). However, a slight spontaneous increase in alizarin red staining over time was also observed in the untreated samples.

To investigate the regulation of sortilin and ALP protein expression during this process, we performed a western blot. Both sortilin and ALP increase over time with OM (Figure 3B). Sortilin shows a significant increase after treatment with OM at day 7 compared with the CM control group (*p* = 0.04) (Figure 3C). ALP is significantly increased in the OM group at day 21 (*p* = 0.02) (Figure 3D). At the same time, we observed an increase in ALP activity on day 21 of osteogenic differentiation (*p* < 0.0001) (Figure 3E). Similar results were observed using murine neonatal chondrocytes (Supplementary Figure S2).

### 2.4. Sortilin Is Upregulated in Senescent Cells

**Figure 4:**
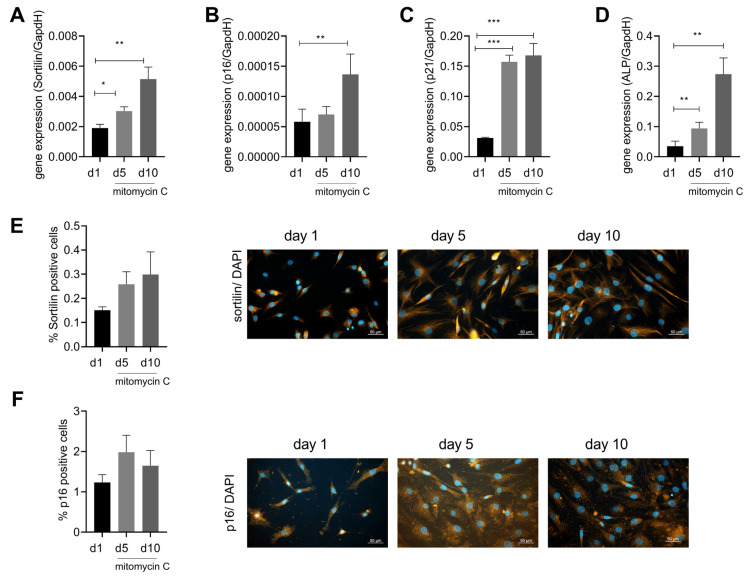
Sortilin is upregulated by senescence. Human chondrocytes were stimulated with mitomycin C for up to 10 days to induce cellular senescence. (**A**) Gene expression of sortilin after 5 and 10 days of treatment with mitomycin C (RM one-way ANOVA F (1.379, 9.656) = 16.88; *p* = 0.0013; *N* = 8). (**B**) Gene expression of p16 after 5 and 10 days of treatment with mitomycin C (RM one-way ANOVA: F (2, 14) = 5.080; *p* = 0.0219; *N* = 8). (**C**) Gene expression of p21 after 5 and 10 days of stimulation with mitomycin C (RM one-way ANOVA: F (1.367, 9.571) = 49.96; *p* < 0.0001). (**D**) Gene expression of ALP after 5 and 10 days of stimulation with mitomycin C (RM one-way ANOVA: F (1.083, 4.331) = 25.42, *p* = 0.0056). (**E**) Sortilin immunofluorescence showing percent sortilin positive cells as a function of duration of stimulation and representative images of sortilin immunostaining at days 1, 5, and 10 (RM one-way ANOVA: F (1.028, 2.057) = 1.077; *p* = 0.4092; *N* = 3). (**F**) p16 immunofluorescence with the percentage of p16 pos. cells depending on the duration of stimulation and representative images of p16 staining at days 1, 5, and 10 (RM one-way ANOVA: F (1.263, 2.527) = 1.153; *p* = 0.399; *N* = 3). All pictures are taken at 400× magnification (scale bar 50 µm). * *p* < 0.05, ** *p* < 0.01, *** *p* < 0.001.

As our previous experiments have linked sortilin to ALP and calcification, we aimed to investigate the regulation of sortilin expression. Cellular senescence is associated with osteoarthritis and might therefore be a trigger for sortilin expression. Furthermore, p16 and p21 were used as senescence markers to verify the induction of senescence using mitomycin C. Interestingly, we observed a time-dependent upregulation of sortilin upon stimulation with mitomycin C (day 5: *p* = 0.02 and day 10: 0.003) (Figure 4A). After 10 days of mitomycin C stimulation, p16 expression was upregulated (*p* = 0.02) (Figure 4B), and after 5 and 10 days of mitomycin C stimulation, p21 expression was significantly increased (day 5: *p* < 0.0001 and day 10: 0.0004) (Figure 4C). Interestingly, ALP was also significantly upregulated during senescence induction after days 5 and 10 (day 5: *p* = 0.0095; day 10: *p* = 0.0109) (Figure 4D). However, in immunofluorescence staining, only a trend for sortilin and p16 increases was observed, which did not reach statistical significance (Figure 4E), although a doubling in the number of senescent cells was observed by p16 stainings (Figure 4F).

## 3. Discussion

After SORT1, the gene coding for sortilin, was linked to increased cardiovascular risk by genome-wide association studies (GWAS) as a possible gene locus, sortilin came into focus as an influencing factor in the development process of age-associated diseases, such as dyslipidemias, atherosclerosis, diabetes mellitus type 2, and also Alzheimer’s disease [12,36,37,38,39]. Sortilin is involved in the secretion and transport of disease-related proteins in the corresponding tissues [17,38,40]. This study reveals that sortilin expression, induced by senescence, impacts the development of OA. Sortilin thus becomes another common factor in the pathogenesis of atherosclerosis and OA.

Previous studies demonstrated a close association between arteriosclerosis and significantly elevated levels of sortilin [17,41]. We observed the same effect for sortilin in OA. An increase in sortilin at protein and gene expression levels was observed in human and murine OA cartilage (Figure 1 and Figure 2), although the severity of OA did not appear to influence sortilin expression.

A previous atherosclerosis study mimicked vascular calcification using human vascular smooth muscle cell (hSMC) stimulation with OM. As the calcification of hSMC increased, the level of sortilin also increased [17]. Similarly, human and murine chondrocytes were treated with OM in this study. An association between sortilin expression and calcified OA chondrocytes was demonstrated in this study. The increase in sortilin expression was accompanied by an increase in ALP activity (Figure 3, Supplementary Figure S2). Human chondrocytes exhibited more rapid calcification and sortilin and ALP increases compared with murine chondrocytes (Figure 3 and Supplementary Figure S2). The human chondrocytes were derived from OA cartilage and therefore originated from an already calcified environment, whereas the murine chondrocytes were isolated from neonatal knee joints. This explains the comparatively faster response of human OA chondrocytes to OM. Already on day 7, a significantly increased level of sortilin at the protein level was measurable in the human chondrocytes (Figure 3). After 21 days of calcification induction, this was followed by significantly increased ALP protein levels and activity (Figure 3). Sortilin is temporally upregulated upstream of ALP. Sortilin may thus be an inducer of ALP in the calcification process of OA. Sortilin-dependent upregulation of ALP has already been demonstrated in atherosclerosis. Sortilin induces both ALP and the calcification of hSMCs and the ejection of calcifying extracellular vesicles [17].

Previous studies demonstrated a close association between cellular senescence and atherosclerosis as well as OA [24,26,28,29,30,42]. One study demonstrated that cellular senescence induces arterial calcification. Increased expression of ALP was found in the senescent hSMC of vessels [31]. In this study, senescent human chondrocytes showed upregulation of sortilin (Figure 4). Sortilin is part of the calcification processes in OA, as described above (Figure 3).

The existing common features of extracellular matrix calcification in atherosclerosis and OA could be extended by this study. Senescence seems to be involved in the calcification process in both diseases. Sortilin provides an important contribution to calcification in OA and atherosclerosis. The exact relationship between sortilin and ALP induction in OA should be the subject of further research.

Our data indicates that sortilin and ALP are upregulated during cartilage calcification, which is associated with chondrocyte senescence and thus might contribute to the pathogenesis of osteoarthritis. Cellular senescence seems to induce sortilin expression.

## 4. Material and Methods

### 4.1. Mouse Model

Knees of 8-week-old (young) and 45-week-old (old) mice were fixed overnight in 4% formaldehyde (Otto Fischar GmbH & Co. KG, Saarbrücken, Germany) at 4 °C, subsequently washed three times with PBS, and decalcified in EDTA at RT for at least 5 weeks. The tissue was embedded in paraffin, and 4-micrometer-thick sections were cut at the microtome.

### 4.2. Human Cartilage

Samples of knee cartilage were collected during the implantation of total knee arthroplasty as well as in unicondylar joint replacement at the Department of Orthopaedic Surgery of the University Hospital Magdeburg. The study was reviewed and approved by the Institutional Review Board (IRB) of the Medical School, Otto-von-Guericke University, Magdeburg (IRB No. 28/20). The patients/participants provided their written informed consent to participate in this study. OA severity was determined radiologically using the Kellgren-Lawrence score (KL-score). The subjects were ranked into mild (KL-score 1–2), moderate (KL-score 3), and severe (KL-score 4) OA. Knee cartilage from people who had died without a history of OA was used as a control. The absence of OA was assessed histologically by the OARSI score.

### 4.3. Chondrocyte Isolation and Cell Culture

Chondrocytes were isolated from the knee joints of 4–5-day-old C57Bl6 wt/wt mice and from human cartilage. Chondrocytes were isolated from murine cartilage tissue by digestion using 3 mg/mL collagenase D (type IV) (Worthington Biochemical Corporation, Lakewood, NJ, USA) for 45 min at 37 °C. The collagenase solution was then diluted 1:6 and incubated with the cartilage pieces over night at 37 °C. Human cartilage samples of patients undergoing knee replacement (KL 3–4) were cut into small pieces and incubated in 1 mg/mL pronase (Sigma-Aldrich, St. Louis, MO, USA) at 37 °C for 30 min. After removal of pronase, overnight digestion was performed with 1 mg/mL collagenase D (type IV) at 37 °C. The suspensions were applied to a cell strainer the next day and centrifuged at 400× *g* for 10 min (Heraeus, Megafuge 16R, Thermo Fischer Scientific, Waltham, MA, USA). The cells were cultured in chondrocyte medium (DMEM High Glucose (Sigma-Aldrich, St. Louis, MO, USA), +10% FCS, +1% penicillin/streptomycin, +1% sodium pyruvate (100 mM)) according to the planned experimental protocol.

### 4.4. Osteogenic Differentiation

Cultured chondrocytes were treated with osteogenic medium (OM; chondrocyte medium including 0.2 mM ascorbic acid, 10 mM glycerophosphate, and 10 nM dexamethasone) for 1, 7, and 21 days to induce calcification. A sample of 3 × 10^5^ cells/well were seeded on sterile 6-well plates (Greiner Bio-One, Kremsmünster, Austria), 1.5 × 10^5^ cells/well on sterile 24-well plates (Greiner Bio-One, Kremsmünster, Austria), and 0.8 × 10^4^ cells/well on sterile 96-well plates (Greiner Bio-One, Kremsmünster, Austria). The OM was changed 3× per week.

### 4.5. Induction of Senescence

Cultured chondrocytes were incubated for 24 h with 200 nmol of mitomycin C (MM, Sigma-Aldrich, St. Louis, MO, USA) in chondrocyte medium at 37 °C [43]. Subsequently, cells were cultured in conventional chondrocyte medium until harvest, on days 5 and 10 after the first stimulation day.

### 4.6. Immunofluorescence Staining

Mouse knees and human cartilage were fixed in 4% formaldehyde (Fischar, Saarbrücken, Germany), embedded in paraffin (Carl Roth GmbH & Co. KG, Karlsruhe, Germany), and the 4-micrometer-thick sections were cut and dried on slides (Thermo Scientific, Waltham, MA, USA). Histological sections were deparaffinized, and antigen retrieval was performed. Chondrocytes simulated with mitomycin C were washed with PBS twice and fixed in 4% formaldehyde for 30 min. For cartilage sections and chondrocytes, epitope blocking was performed in 4% bovine serum albumin fraction V (BSA; Sigma-Aldrich, St. Louis, MO, USA). The primary antibodies used were mouse-specific anti-sortilin polyclonal goat antibody (1:500; R&D systems, Bio-Techne Corporation, Minneapolis, MN, USA), anti-sortilin/NT3 polyclonal rabbit antibody (1:500; Abcam, Cambridge, MA, USA), anti-p16-INK4A polyclonal rabbit antibody (1:200; Abcam, Cambridge, MA, USA), and mouse-specific anti-p16INKA4 polyclonal rabbit antibody (1:200; ProteinTech, Rosemont, IL, USA) incubated overnight at 4 °C. The fluorescently labeled secondary antibodies, i.e., Anti-goat Alexa 555 and Anti-rabbit Alexa 555 (1:500; Invitrogen, Carlsbad, CA, USA), were incubated species-specifically for 60 min at 25 °C. Sections were covered with ROTI-Mount FlourCare DAPI (Carl Roth GmbH u. Co. KG, Karlsruhe, Germany), and immunofluorescence was detected microscopically (Axio Observer, Axiocam 702 mono, HXP 120V, Carl Zeiss, Jena, Germany).

### 4.7. RNA Analysis

For RNA extraction, human cartilage samples were minced and dissolved in 1 mL of Trizol (Qiagen, Hilden, Germany). The RNeasy Micro Kit (Qiagen, Hilden, Germany) was used according to the manufacturer’s protocol. The mitomycin C-treated chondrocytes were harvested, and RNA extraction was performed using 500 µL of Trizol according to the manufacturer’s protocol.

The amount and purity of the isolated RNA were determined photometrically, and 500 ng of RNA was reverse-transcribed into cDNA via reverse transcription using the High-Capacity cDNA Reverse Transcription Kit (Thermo Fischer Scientific, Waltham, MA, USA). Gene expression was assessed by quantitative real-time polymerase chain reaction (RT-PCR) using the Applied Biosystems QuantStudio6 Flex Real-Time PCR System (Thermo Fischer Scientific, Waltham, MA, USA). RT-PCR was performed using PowerTrack SYBR Green Master Mix (Thermo Fischer Scientific, Waltham, MA, USA) according to the manufacturer’s protocol and the following primers: Sortilin (forward AATGGCCTGTGGGTCCAA and reverse AGGTCAGCTTTGCAGGAGCC), p16 (forward CAACGCACCGAATAGTTACG and reverse ACCAGCGTGTCCAGGAAG), p21 (forward GGAGACTCTCAGGGTCGAAA and reverse CTTCCTGTGGGCGGATTA), and GAPDH served as housekeeper (forward CCCACTCCTCCACCTTTGAC and reverse AGGTCAGCTTTGCAGGAGCC). Absolute quantification was carried out using standard curves. Target gene expression was normalized to Glyceraldehyde-3-Phosphate Dehydrogenase (GAPDH).

### 4.8. Calcification

Alizarin Red S staining was performed on days 1, 7, and 21. After discarding the medium, cells were washed twice with 100 µL PBS (Sigma-Aldrich, St. Louis, MO, USA), fixed in 100 µL 4% formaldehyde for 30 min, and then washed twice with 100 µL distilled water (dH_2_O). Staining was performed with 100 µL of 2% Alizarin Red S solution (2 g Alizarin Red S (Sigma-Aldrich, St. Louis, MO, USA) in 100 mL of dH_2_O pH: 4.1–4.3) for 45 min in the dark. After 2 times washing with 100 µL dH_2_O, destaining was performed with 50 µL cetylpyridinium chloride (Sigma-Aldrich, St. Louis, MO, USA). After 5–10 min of incubation, 50 µL of the solution was transferred to a 96-well microassay plate (Sarstedt AG & Co. KG, Nümbrecht, Germany), and absorbance was measured at 560 nm (Infinite F200 Pro, Tecan Group AG, Switzerland).

### 4.9. Alkaline Phosphatase

ALP activity was measured in cell lysates using a colorimetric assay according to the manufacturer’s protocol (ab83369, Abcam, Cambridge, MA, USA). Cells were harvested from sterile 24-well plates on days 1, 7, and 21 of treatment, resuspended in 50 µL assay buffer, and stored at −80 °C until activity determination. p-nitrophenyl phosphate (pNPP) was used as the enzyme substrate, which appears yellow after dephosphorylation by ALP. The assay was performed on a 96-well plate. After 60 min of light-protected incubation at 25 °C, the reaction was stopped by adding Stop-Solution, and absorbance was measured at 405 nm (Infinite F200 Pro, Tecan Group AG, Switzerland).

### 4.10. Western Blotting

Sortilin and ALP in chondrocytes were detected semiquantitatively by Western blotting. After the harvest of cultured cells, protein extraction was performed in 40 µL of RIPA lysis buffer, followed by storage at −80 °C. A 15-microgram sample of protein was mixed with 1/5 sample buffer (glycerol (30%); SDS (10%), 2-mercaptoethanol (5%), 1 M Tris-HCL pH 6.8, and 2 mg bromophenol blue (0.02%)) and denaturation was performed for 5 min at 95 °C. Proteins were separated at 120 V in a 10% polyacryamide gel and then transferred to a nitrocellulose membrane (BioRad Laboratories, Hercules, CA, USA) at 350 mA. Membranes were blocked with 5% BSA (Sigma-Aldrich, St. Louis, MO, USA) in Tris-buffered saline buffer (TBS) for 60 min at 25 °C. The membrane was then incubated with anti-sortilin/NT3 polyclonal rabbit antibody (1:1000; Abcam, Cambridge, MA) or anti-ALP (Tissue Non-Specific) polyclonal rabbit antibody (1:1000; GeneTex Inc., Irvine, CA, USA) or anti-GAPDH monoclonal mouse antibody (1:1000; Cell Signailing Technology, Danvers, MA, USA) at 4 °C overnight. This was followed by 60 min of incubation with horseradish peroxidase (HRP)-conjugated goat anti-mouse antibodies (1:10,000; Santa Cruz Biotechnology; Santa Cruz, CA, USA) and goat anti-rabbit antibodies (Cell Signailing Technology, Danvers, MA, USA). Proteins were visualized and detected using the Clarity Western ECL Substrate and Imaging System (ChemiDoc MP, BioRad Laboratories, Hercules, CA, USA).

### 4.11. Statistics

All data were presented as mean ± SD. Unpaired t-tests were used for analyzing data comparing two groups for statistical significance. Data with more than two groups were analyzed by a repeated measures one-way analysis of variance (ANOVA) or a one-way ANOVA followed by a Dunnett’s test as a post hoc test in the case of a statistically significant ANOVA result. Data that were investigated for more than one parameter were analyzed using a two-way ANOVA followed by a Sidak’s test as a post hoc test in the case of a statistically significant ANOVA result. A Shapiro-Wilk normality test was performed to identify parametric or non-parametric data distributions. GraphPad Prism V.6.00 for Windows (GraphPad 8 Software, La Jolla, CA, USA) was used. Statistical significance was determined at a level of *p* ≤ 0.05.

## Data Availability

The raw data supporting the conclusions of this article will be made available by the authors without undue reservation.

## Supplementary Materials

The following supporting information can be downloaded at: https://www.mdpi.com/article/10.3390/ijms241512343/s1.
