# Supplementary material for: Sortilin Is Upregulated in Osteoarthritis-Dependent Cartilage Calcification and Associated with Cellular Senescence
Source: Int J Mol Sci. 2023 Aug 2;24(15):12343. doi: 10.3390/ijms241512343 (PMC10418692; doi:10.3390/ijms241512343)
Supplement: Supplementary file 1 [file ijms-24-12343-s001.zip › ijms-2540059-supplementary.pdf]

Supplementary Materials

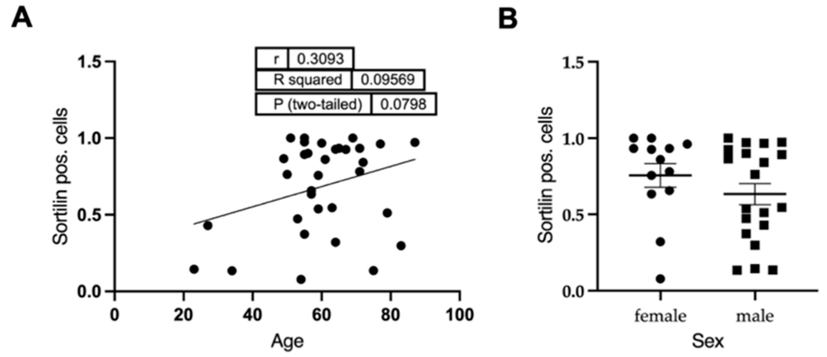

Supplementary Figure S1. Age-and sex-correlation of sortilin expression in human cartilage. IF staining of sortilin on human cartilage. (**A**) Sortilin-pos. cells as a function of age. There is no correlation between patient age and sortilin. (**B**) Sortilin-pos. cells as a function of sex. There are no significant differences for sortilin in male and female subjects.

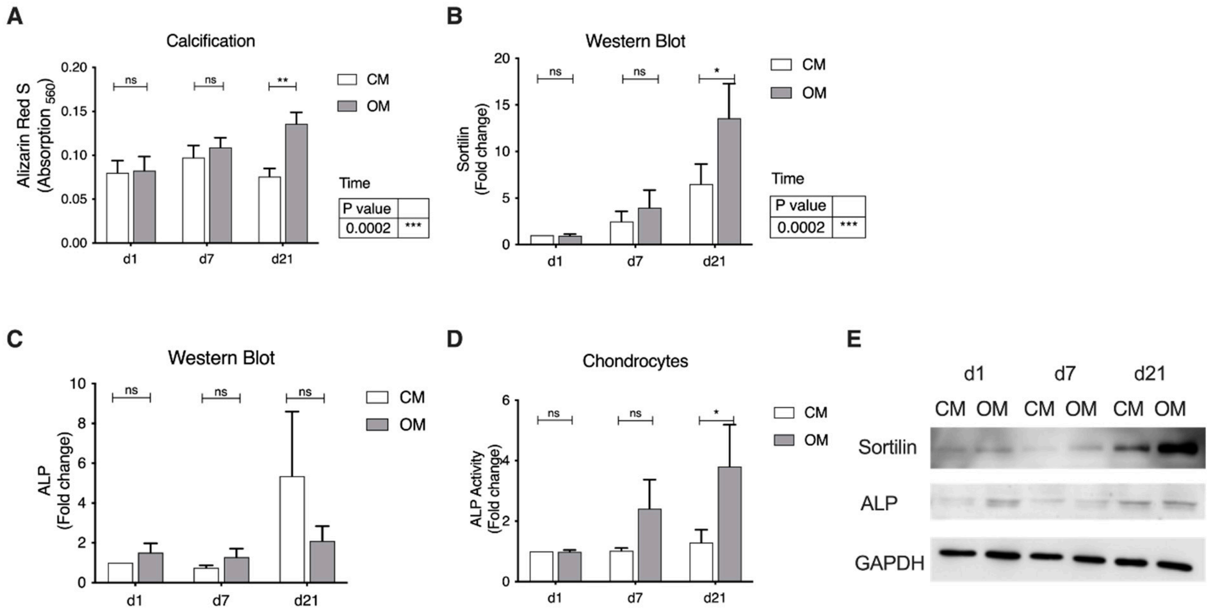

Supplementary Figure S2. Sortilin and ALP are upregulated in osteogenic differentiated murine chondrocytes. Murine chondrocytes were treated for up to 21 days with osteogenic medium (OM) to induce calcification and chondrocyte medium (CM) was used as a control. (**A**) Alizarin Red S staining of chondrocytes after treatment with CM and OM to measure calcification. Treatment with OM results in significantly increased calcification at day 21, compared with control (CM). Furthermore, there is a significant increase in calcification over time ($p = 0.0002$). (**B** and **D**) Western blot of sortilin and ALP after osteogenic differentiation. (**B**) Sortilin is significantly increased at day 21 (OM) compared to control (CM). Over time, there continues to be a significant increase in sortilin ($p = 0.0002$). (**D**) ALP shows no significant changes at the protein level. (**E**) Representative Western Blots of Sortilin and ALP. (**C**) ALP activity assay of CM and OM chondrocytes. At day 21, ALP activity of OM chondrocytes is significantly increased to CM chondrocytes. ns: not significant; * $p < 0.05$, ** $p < 0.01$.
